# Yarning about foot care: evaluation of a foot care service for Aboriginal and Torres Strait Islander Peoples

**DOI:** 10.1186/s13047-022-00524-9

**Published:** 2022-04-04

**Authors:** Matthew West, Sean Sadler, James Charles, Fiona Hawke, Sean Lanting, Shannon E. Munteanu, Vivienne Chuter

**Affiliations:** 1 Discipline of Podiatry, University of Newcastle, Ourimbah, NSW 2258 Australia; 2 First Peoples Health Unit, Griffith University, Gold Coast, QLD 4215 Australia; 3 Discipline of Podiatry, School of Allied Health, Human Services and Sport, La Trobe University, Melbourne, Victoria 3086 Australia; 4 La Trobe Sport and Exercise Medicine Research Centre, School of Allied Health, Human Services and Sport, La Trobe University, Melbourne, Victoria 3086 Australia; 5 School of Health Sciences, Western Sydney University, Campbelltown, NSW 2560 Australia

## Abstract

**Background:**

Aboriginal and Torres Strait Islander Peoples have high rates of diabetes-related foot disease including foot ulcer and amputation. There has been limited evaluation of foot care services for Aboriginal and Torres Strait Islander Peoples. This project aimed to evaluate an Aboriginal and Torres Strait Islander foot care service (the Buridja Clinic) for prevention and management of diabetes-related foot disease embedded in a university podiatry program from a Community perspective using culturally appropriate methods.

**Methods:**

This mixed-methods study took place from March 2018 to April 2021 in the Buridja Clinic on the Central Coast of New South Wales, Australia, and included an audit of occasions of service (March 2018 to March 2020), and review of the Buridja Clinic via research yarns with Aboriginal and Torres Strait Islander clients of the clinic and a written 10-item customised clinic feedback survey. Research yarns were transcribed and analysed thematically. Descriptive analysis of quantitative occasions of use and survey data was undertaken, with the open-ended survey responses thematically analysed.

**Results:**

Total occasions of service across the review period was 548, with a total of 199 individual clients treated. Most common service types were general treatments (nail and skin care) and diabetes assessments. Nine participants who attended the Buridja Clinic were recruited to the two research yarns. An additional 52 participants who attended the clinic completed the customised clinic feedback survey. Specific clinic design elements, including yarning circles and group booking as well as student placement, were identified as strengths of the clinic. Participants reported difficulty with transport and restricted opening hours and encouraged increased Community engagement by clinic staff.

**Conclusion:**

Evaluation of a foot care service for Aboriginal and Torres Strait Islander Peoples embedded in a university-based podiatry program demonstrated that the incorporation of specific service design elements, including yarning circles and group appointments as well as student placements, encouraged ongoing Community engagement with the service. Participants reported improved foot health, greater foot and self-care knowledge, and overall better general health and management as a result of attendance to the clinic. Consideration needs to be given to addressing limited access to transport and flexible operating hours when establishing similar services.

**Graphical abstract:**

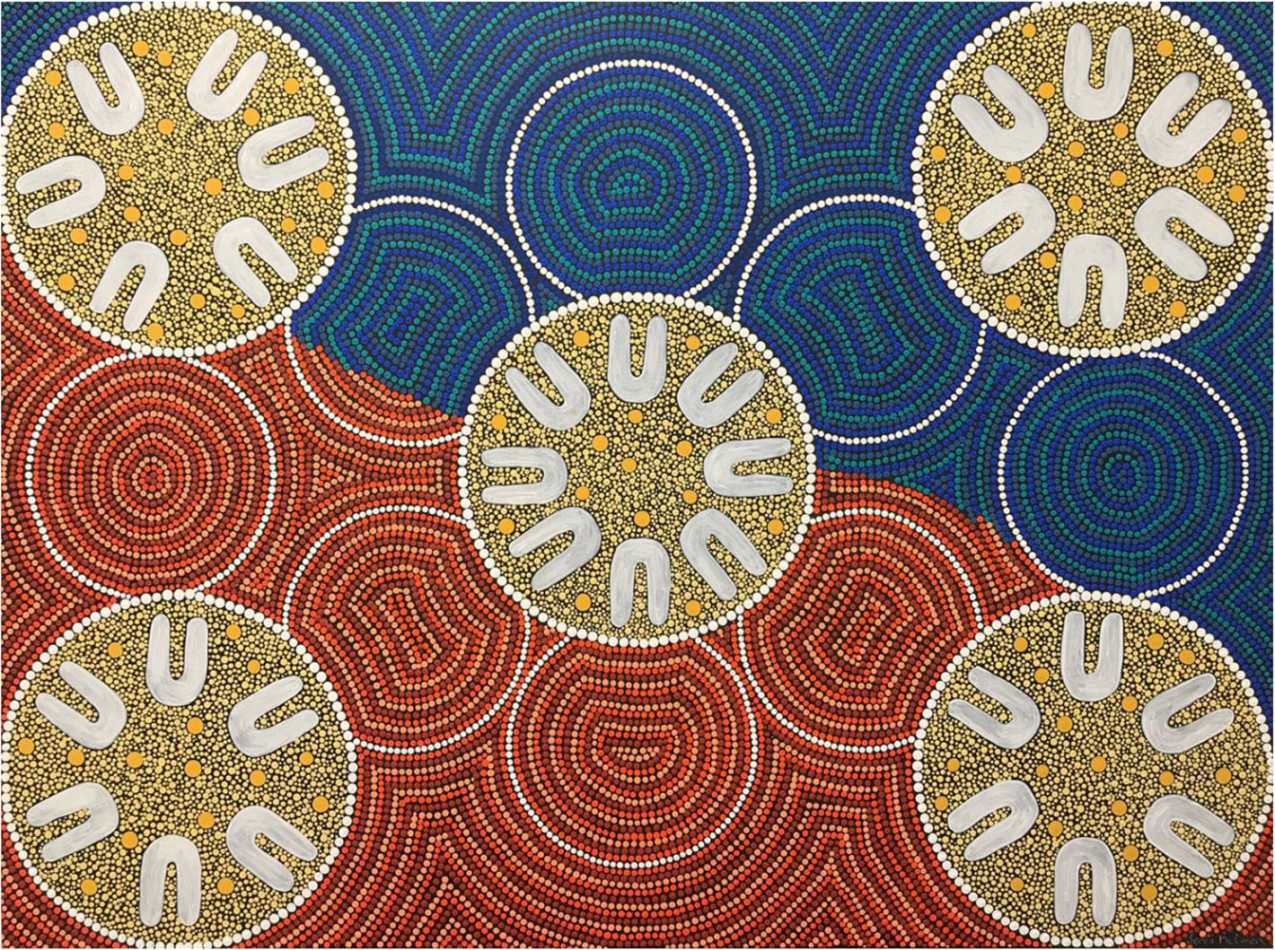

Artist Jenni McEwen (Bundjalung) lives on Darkinjung Country. The story of her art shows people sitting in yarning circles sharing knowledges but looking outwards to connect with Country too, the Ochre of Wiradjuri Country around Wellington, and the Blue of Darkinjung Country around Central Coast. These are locations where podiatry service provision takes place. These are locations where everyone is sharing and learning. Students, teachers, patients, non-Indigenous people, and First Nations people, learning from each other, and learning from Country.

**Supplementary Information:**

The online version contains supplementary material available at 10.1186/s13047-022-00524-9.

## Introduction

Diabetes is associated with a life-time incidence of foot ulcer of up to 30% and is the leading cause of lower limb amputation [1]. Of people undergoing an amputation, half will die within the subsequent five years [2]. Aboriginal and Torres Strait Islander populations are known to have disproportionately high rates of diabetes, with the age-standardised disease rates up to four times that of non-Indigenous Australians [3]. Consistent with this, diabetes-related complications are responsible for almost 70% of preventable hospitalisations for chronic conditions for Aboriginal and Torres Strait Islander Peoples, with the likelihood of diabetes-related foot disease (DFD) increased by five-fold relative to non-Indigenous Australians [4, 5].

The lack of availability of culturally safe foot care services, and the effects of ongoing dispossession and distrust of Western healthcare systems which are linked to institutional and historical racism, have been identified as key contributors to worse DFD in Aboriginal and Torres Strait Islander Peoples [6–8]. Despite the urgent need for effective preventative foot care in this population, our data indicate low levels of engagement with existing preventative care services, and limited evaluation of Aboriginal and Torres Strait Islander specific foot care services [9, 10]. Evidence shows rates of access for wound care services for Aboriginal and Torres Strait Islander people are almost double those of non-Indigenous people, while use of preventative services including neurovascular assessment and general treatment are consistently lower [10].

In 2018 the Buridja Clinic was established in the Discipline of Podiatry at the University of Newcastle (UON) to deliver a culturally safe foot care service focused on DFD prevention. In being culturally safe, the clinic was designed to create an environment that recognises and actively engages with the spiritual, physical, social, and emotional world view of Aboriginal and Torres Strait Islander people, thereby creating a clinical experience which is conducive to, and supportive of, the specific needs of this Community [11]. Additionally, the approach to management of patients within the clinic is one that recognises the importance of Country, culture, language, ceremonies, family, and Community for Aboriginal and Torres Strait Islander people. The clinic was developed in consultation with the local Aboriginal and Torres Strait Islander Community, community health care providers and Aboriginal organisations including Darkinjung Local Aboriginal Land Council and Yerin Aboriginal Medical Service, and provides foot assessment, foot care and health promotion to the Aboriginal and Torres Strait Islander Community free of charge. The clinic is staffed by an Aboriginal Podiatrist, Aboriginal Health Worker and UON podiatry students. All students attend a minimum of three days at the Buridja Clinic over their final year of their studies. The Buridja Clinic has now run since 2018 as a one day per week Community clinic, providing over 500 instances of foot assessment and management, as well as health care education provided via a weekly yarning circle. The clinic is hosted in an established podiatry clinical facility at Wyong Hospital and offers a limited transport service to and from the clinic. In terms of Western measurements of health service success including occasions of service delivered, this represents an effective program. However, ongoing dialogue with end-users (clients of the service, Community members and health care providers) has identified that these data fail to provide any measure of Aboriginal perspectives of program success. As a result, the components of the program that, from a participant and Community perspectives, are successful in maintaining long-term engagement with, and participation in, the service are indistinguishable. Clarifying these aspects of the program, through investigation incorporating more culturally appropriate approaches, is essential to developing an effective clinical model that is translatable across Communities. More broadly this represents a largely un-researched aspect of Aboriginal and Torres Strait Islander health care delivery, and one that has been poorly recognised in the development of foot care strategies to combat the significant over-representation of foot complications in this population [9].

Previous research has advocated for research in Indigenous populations to use methods which value the knowledge and experience of Indigenous Peoples [12–14]. Yarning is an Aboriginal and Torres Strait Islander way of storytelling, teaching and learning that is a collaborative dialogical progression [12]. Yarning as a research method is defined as being a conversation with a purpose (with similarities to a semi-structured interview) that applies storytelling and actively listening to collecting information. It requires researchers to build an accountable relationship with participants and to allow storytelling that does not necessarily adhere to a research plan or research question(s) and to identify common threads that inform the research topic [13, 14]. Yarning has previously been used to investigate Community perspectives on Aboriginal healthcare programs [15, 16] and has integrated into the Buridja Clinic as a means of developing a mutual understanding between health care providers and clinic clients, and gives a voice to Community about foot health and foot care. This method of research offers an effective means to undertake a culturally appropriate evaluation of the Buridja Clinic, focusing on Aboriginal perspectives of program success and the impact of the clinic on holistic well-being.

The aim of this research was to evaluate the Buridja Clinic from an Aboriginal and Torres Strait Islander perspective using culturally appropriate research methods. Specifically the research aimed to determine the relative success or otherwise of the Buridja Clinic from the perspective of Aboriginal and Torres Strait Islander clients as end-users of the service, and investigate which areas of the clinical model were effective or required improvement.

## Methods

This mixed-methods study took place in the Buridja Clinic at the UON student podiatry clinic at Wyong hospital, Central Coast, New South Wales, Australia. This study included an audit of occasions of services collected between March 2018 and March 2020, a written 10-item customised clinic feedback survey, and a qualitative review of the Buridja Clinic via research yarns. The use of a mixed method approach enabled the researchers to quantify the outcomes of the Buridja Clinic from an objective and Western academic viewpoint in relation to occasions of service delivery, and from a Community perspective through yarning sessions allowing exploration of Aboriginal Community perspectives of the Buridja Clinic. Using a combination of these methods facilitated exploration of the interrelationship between clinic use and patient reported experiences. The study was approved by the University of Newcastle ethics committee (H-2018-0035) and the Aboriginal Health and Medical Research Council (1376/18).

### Clinical audit

Data relating to occasions of services (total number and service type) were measured across a two-year period between March 2018 and March 2020. During this time the clinic operated during the University semester with a limited service provided between semester dates that was operated by university staff. Data were extracted retrospectively from University clinical records by MW using manual coding of occasion of service type. Due to the clinic operating as a drop-in service, non-attendance data were not collected.

### Customised feedback survey

The customised survey was offered to all attendees at the clinic to be completed and submitted anonymously. The survey included three questions relating to number of attendances, types of services accessed, and referral source (e.g. word of mouth, health practitioner referral), six questions relating to their experiences at the clinic with staff, students and the services, and one open ended question for additional feedback about any aspect of the clinic. The six questions relating to their experience at the clinic required respondents to rate aspects of the clinic on a 5-point Likert scale ranging from ‘very good’ to ‘very poor’, ‘very likely’ to ‘very unlikely’ or ‘very easy’ to ‘very difficult’ (Supplementary file 1). To quantify the responses, the most positive response options (i.e. very good, very likely, and very easy) were allocated a score of 5, while the least positive were scored at 1 (i.e. very difficult, very unlikely, and very poor). Survey data were entered into Microsoft Excel and then exported to the Statistical Packages for Social Sciences (SPSS) (version 25.0 Chicago, Illinois, USA) for analysis and frequencies were calculated. Open-ended responses were manually collated, categorised and coded by two researchers. From this key themes and sub-themes were identified. The thematic analysis was conducted by an Aboriginal researcher (MW) and crossed-checked by a second researcher (VC).

### Research yarns

For research yarns, recruitment of participants was conducted using purposive and selective sampling from clients of the Buridja Clinic. Research yarns were conducted in the same space as the weekly yarning circles at the UON podiatry clinic. The research yarns explored the Community perspective on the Buridja Clinic and were guided by two key aims developed with the Community members. These were to explore:

1. The researchers' and participants' shared understanding of the elements that are important to creating a successful foot health program for Aboriginal and Torres Strait Islander Peoples.
2. The role of the Buridja clinic in participants’ own perspectives of their foot health and overall well-being.

Using these aims the research yarns discussed essential elements in the clinical service model that were compatible or incompatible with Community expectations and considered successful or not, and explored Community perspectives of the program, including how program and participant success should be evaluated. Secondly, the research yarns explored the participant perceptions regarding if, and how, the Buridja Clinic had affected their self-perceived foot health and overall well-being. The research yarns were led by an Aboriginal researcher (MW). Prompts were not used as it has been previously identified that maintaining adherence to the research question or topic can prevent relevant storytelling that does not obviously adhere to research questions or academic concepts [15].

Yarning circles were recorded, transcribed verbatim and independently analysed and coded by two researchers including an Aboriginal researcher (MW and VC). Data were then categorised and coded through several iterations into key themes and sub-themes, identifying similarities and differences within and between the yarning with any emerging patterns identified. Generated codes were refined from recurring responses and developed into themes for analysis. Over several iterations, new coded material and emerging patterns were discussed at regular intervals and regrouped into key themes and sub-themes, identifying similarities and differences within and between the yarning. These themes were then cross-checked by an Aboriginal researcher (MW) to identify areas of potential bias and to determine the reliability of the data interpretation [17, 18]. Prior to data analysis all participants were offered the opportunity to review their yarning sessions and verified their data.

## Results

Clinical audit: Over the time frame of March 2018 to March 2020, 58 clinical sessions were run in the Buridja Clinic and 199 individuals attended. Total occasions of service across the two years was 548. Most common service types were general treatments and diabetes assessments (comprising vascular and neurological testing, and risk classification in a person diagnosed with diabetes). A breakdown of occasions of service is shown in Table 1.

**Table 1:** Occasions of service, n  (% of all service types)

Service type	2018	2019/20^a^	Total
General treatments, n	53 (21.1)	72 (24.2)	125
Vascular assessments, n	28 (11.2)	27 (9.1)	55
Neurological assessments, n	25 (10.0)	23 (7.7)	48
Diabetes assessments, n	52 (20.7)	62 (20.9)	114
Biomechanical assessments, n	15 (6.0)	22 (7.4)	37
Orthotic prescription, n	10 (4.0)	12 (4.0)	22
Footwear prescription, n	44 (17.5)	56 (18.9)	100
Nail surgery, n	2 (0.80)	2 (0.7)	4
Group education sessions, n	22 (8.8)	21 (7.1)	43
Total occasions of service	251	297	548

^a^Data from 2019 to March 2020 combined

### Customised survey responses

Fifty-two participants completed the customised survey. The mean (SD) number of clinic attendances per participant was 3.9 (2.1). Approximately 54% of participants (*n* = 28) became aware of the service from word of mouth, 19% (*n* = 10) from referral from another health practitioner, 12% (*n* = 6) from advertising and 15% (*n* = 8) from other sources. Other sources most frequently included Community events where the clinic held outreach services including National Aborigines and Islanders Day Observance Committee (NAIDOC) week and Coast Connect days. The most common reasons for visiting the clinic were for general foot care (69%, *n* = 36), diabetes assessment (65%, *n* = 32), diabetes education (23%, *n* = 12), footwear referral (21%, *n* = 11), foot orthoses (10%, *n* = 5) and nail surgery (2%, n = 1). Results for questions 4–9 of the survey relating to participants rating their experience of different aspects of the clinic are shown in Table 2.

**Table 2:** Ratings of participant experience with the Buridja clinic

Survey component	Responses (scores 1–5)				
	1	2	3	4	5
Clinic access, n (%) *(5 very easy, 4 easy, 3 neutral, 2 difficult, 1 very difficult)*	0	0	12 (23)	29 (56)	11 (21)
Availability of foot care services, n (%) *(5 very good, 4 good, 3 neutral, 2 poor, 1 very poor)*	0	0	8 (15)	31 (60)	13 (25)
Experience with students, n (%) *(5 very good, 4 good, 3 neutral, 2 poor, 1 very poor)*	0	1 (2)	3 (6)	28 (54)	20 (38)
Experience with staff, n (%) *(5 very good, 4 good, 3 neutral, 2 poor, 1 very poor)*	0	0	0	24 (46)	28 (54)
Quality of care, n (%) *(5 very good, 4 good, 3 neutral, 2 poor, 1 very poor)*	0	0	0	12 (23)	38 (73)
Likelihood of recommending the clinic, n (%) *(5 very likely, 4 likely, 3 neutral, 2 unlikely, 1 very unlikely)*	0	0	0	13 (25)	39 (75)

The opened-ended response for feedback identified three main themes, two of which related to access to the clinic and one to clinic services. The two themes relating to clinic access related to transport and scheduling. Specifically, participants identified a lack of onsite parking and poor public transport as hindrances to them attending the service. In addition, the restricted hours of the clinic (one day a week) was identified as preventing family members attending the clinic. Participants noted a preference for an out-of-hours or weekend session as well to allow more family members who were working or at school to attend, as well as the option for the clinic to run at additional Community sites (outreach clinics).

### Research yarns

Two research yarns were conducted which ran for approximately 40 minutes and included a group of five participants and a group of four participants. All participants were clients of the clinic and had regularly attended the clinic over a period of 24 months (more than six occasions of service each). Participants were six women and three men (including three Community Elders), age ranged from 35 years to 85 years, with the majority of the participants (6 out of 9) having an existing diagnosis of diabetes.

Congruence was observed in participant responses across the two yarning circles with no new themes raised in the second yarn. An overview of the themes raised in relation to program success and self-perceived foot health and well-being is provided in Table 3 below.

**Table 3:** Overview of themes raised in research yarns

Measures of program success	Foot health and overall well-being
Students’ preparedness for placement	Improved foot health
Student interactions and engagement	Change in self-care habits, general and foot-related
Cultural sensitivity	Change in footwear use
Clinical environment	Increased use of other health services
Inclusion of culturally safe elements (yarning circles)	Improved health literacy
Presence of Aboriginal health care providers	
Community engagement by non-Indigenous staff	

### Measures of program success

Overall, participants reported that they were satisfied with the design of the clinic. Successful elements of the clinic related to interaction with the students, the clinical environment and improved health literacy with recognition of the clinic as helping to reduce their fears of poor health outcomes. There was a strong consensus among participants that the students involved were an integral part of the success of the clinic, and, that this increased their own engagement with the service. Themes relating to the positive role of students in the service included identification of a high level of preparedness for undertaking placement and their active engagement with the clients.

*“The pre-course material that were introduced to them before meeting Community members is obviously very well structured because that was evident in how they conducted themselves, how they approached the Community members, and those conversations and how they engaged. How they treated me and the other people in the room there was a lead up to that for them to be ready to come into that room. So obviously that pre-work must be very good.”* [P4]

It was specifically noted that although the individual providing treatment changed many times, participants felt the student body was responsive to the cultural needs of the Community and recognised a role for themselves in the student training.

*“I found the students to be respectful, they were culturally sensitive to us as Aboriginal people which I found to be really good … They wanted to learn, they’d ask a question about your Aboriginality, where you come from and who you were, and that was really interesting because they were learning about us as Aboriginal people.”* [P8]

*“The students, all the ones that come, they’re all such beautiful people. And every time I go there would be different ones there and they were all lovely.”* [P3]

*“It’s breaking down the stereotypes … giving us the chance to talk to the students and teach them.”* [P5]

Design aspects of the clinic model, such as yarning circles, group appointments, and education sessions, were identified by participants as making them feel more likely to attend and encouraged Community engagement.

*“It was enjoyable going and meeting up with different people that I knew also and getting to have a yarn to the students and tell them about my life.”* [P6]

Participants also identified the role of the clinic in helping them understand their health, and giving them knowledge and understanding to better manage the condition. Participants clearly linked their own health concerns with experiences of their family members. This was particularly profound from family members who had diabetes. Of note was the universally negative impact diabetes had on the family members of participants. Family members of participants who had suffered negative outcomes served to motivate participants to engage in prevention strategies.

*“I’ve had sisters be diabetics and one of my sisters passed away a couple of years ago through diabetes. She was in hospital and going through all sorts of things, but she couldn’t walk around that much because of her diabetes, I like coming here to get everything checked.”* [P3]

*“I have a brother with bad diabetes, and he has lost a toe and there no way in the world that I want to be in that position. I hope I never get in that position. This clinic has helped me understand the importance of my diet and how to maintain that and to have regular check-ups and ensure I stick to that because of the diabetes and having a number of people pass because of diabetes and obesity so I’m a lot clearer now about how to maintain my diabetes and my healthy feet, as well as a healthy diet.*” [P1]

The importance of engagement in a Community context more broadly outside of the clinical environment was consistently identified. A strength of the clinic was the presence of an Aboriginal Podiatrist and Aboriginal Health Worker from the local Community.

*“It’s good to have the clinic, it’s good to be able to come here and get sorted out, to have people from our Community here and they tell us where we need to go or talk to the doctor.”* [P7]

Community engagement also related to non-Indigenous staff who had an ongoing presence in the clinic and ensuring they are more widely recognised in the Aboriginal and Torres Strait Islander Community. The importance of people in the service being linked to that service and being known in the Community was highlighted.

*“For me some of the non-Indigenous staff that [work with the team at the clinic] is a good way for them to engage a lot better with the Community is to be seen out in Community maybe not so much in the clinic. But to come along to a local Community event and encourage the podiatry program and what it’s about and the importance of the podiatry program, more people will come if they get to know them better.”* [P2]

*“Advertising, definitely advertising putting it out there that whether they’re black feet or white feet they need to be looked after. With advertising, ‘we walk together’ which is one of the things we talk about in reconciliation. So when we have our events even like with NAIDOC.”* [P1]

### Self-perceived foot health and general health

The complex nature of many chronic illnesses combined with the central role of self-care/self-management in the long-term proactive management of such conditions was recognised by participants in both groups. Further to this, understanding the improvement in health literacy and self-care knowledge among the group as they participated in the program was evident in the comments they expressed.

*“It opened my eyes about neuropathy. I think with diabetes we’re more worried about blood pressure and sugars and stuff like that and not so much the damage that’s happening down lower in our feet.*” [P2]

Self-reported improvement in foot health was also noted and changes in self-care behaviour in response to attending the clinic. These were consistently reported in relation to footwear changes to try to prevent foot complications.

*“It gave me greater understanding of that foot care knowledge everybody should have as a diabetic person and down the roads of wearing good shoes, good quality shoes.”* [P6]

*“I know what I should be looking for in a shoe now.”* [P9]

*“They helped me find shoes that were good but not like the real expensive ones because they are too much.’* [P3]

Changes in knowledge of how to treat foot problems and prevent additional complications were also acknowledged including daily foot checks and emollient use.

*“I had really, really bad feet...now they are completely cleared up because of knowing how to maintain and look after them.”* [P1]

*“To check my feet for cuts and little things so you don’t get infections because usually you just don’t bother.”* [P6]

*“Overall for me to manage my diabetes and keep it at fours and fives [blood glucose concentration level, mmol/L] means then I have good feet …* A*nd the moisturising of the harder skin on your feet to make it better.”* [P8]

Participants also reported changes in their health care seeking behaviour. This included increased likelihood of visiting health care providers for preventative health care. For example, where clients had not directly been referred on, they were motivated to engage more with their primary health physician.

*“I think the diabetes management is a bit better than it was, and I need to see my doctor and get up to date medications.”* [P2]

In addition to greater motivation to seek preventative health care, participants also reported increased likelihood of them advising other family members to use preventative health care, and increased likelihood of them attending other health practitioners they were referred to from the Buridja Clinic.

*“I had one test and one leg was warm and one cold and a letter was written to my doctor to check that out and it was discovered I had a baker’s cyst and they did a full ultrasound on it.”* [P1]

*“They were worried about my skin at the clinic and sent me to have it checked and I had to get some stuff cut-off. Now the whole family has gone and got checked.”* [P9]

## Discussion

This study is one of the first to investigate Aboriginal and Torres Strait Islander Peoples' perceptions of success of a podiatry service designed for Aboriginal and Torres Strait Islander Peoples alongside review of service utilisation [9]. The clinical audit data extracted as part of this research demonstrated sustained engagement of Community members with the service over the period of the audit. The high use of diabetes assessments and general treatment is consistent with high rates of diabetes in the Community [19]. This also reflects DFD prevention through preventive treatment and improved self-care capacity being a priority for the local Aboriginal and Torres Strait Islander Community.

Further to evidence of consistent service use that was aligned with Community expectation of the Buridja Clinic, this research has provided insights into elements of service that contribute to perceived success by the Community. Consistent with previous research this present study supports the essential role of local Community involvement in the design and development of the clinic and in staffing of the clinic and the need for a shift away from traditional service model structure to create a culturally safe foot care service [9]. Interestingly, our study findings also demonstrated a positive response by Community members to the integration of undergraduate podiatry students into the clinical service delivery model. The strong positive feedback on this element of the Buridja Clinic was noteworthy, with this seen by participants as both an opportunity for them to generate change in perception of Aboriginal and Torres Strait Islander Peoples and health outcomes, and part of their own role in the clinic. This may have contributed to an increased sense of ownership of, and engagement with, the clinic, which is essential for success.

Lack of access to culturally safe general health care, and limited service delivery with related health workforce pressures, including difficulty recruiting health graduates to rural and regional positions, and limited cultural capability in health care delivery, are key contributors to poorer health outcomes for Aboriginal and Torres Strait Islander Peoples [20]. There has been very limited evaluation of the effectiveness of university-based training on students’ cultural capability outcomes for health graduates in any form, but particularly in relation to clinical placements [9, 21]. Studies of the effectiveness of cultural awareness and cultural capability programs in Aboriginal and Torres Strait Islander populations and Indigenous populations globally have shown inconsistent results [21–23]. There is evidence that inclusion of programs focussed on Indigenous health can increase student preparedness for undertaking work with Indigenous peoples, and may generate increased commitment to improving health outcomes for this population in the future [23, 24]. Beyond this, to the authors’ knowledge, there is a paucity of evidence evaluating the involvement of health students in delivering health services for Aboriginal and Torres Strait Islander Peoples from a Community perspective. Our findings of student placement in the Buridja Clinic as being a strength of the service highlights the potential for university-based health programs to improve the cultural capability of graduates and produce enduring change in the capacity of health care workforce to provide culturally safe care.

The outcomes of our study highlight the importance of a multifaceted approach to Community engagement to support uptake of health care and health education services in Aboriginal and Torres Strait Islander Communities. Increasing Community engagement of staff outside the health service via general Community events was identified by participants as a way to continue to increase the overall Community engagement with the clinic. While Community consultation and client consultation is central to the ongoing management of the Buridja Clinic, there is capacity for much wider consultation and engagement with the broader Community that will be important for future growth of the service and to improve Community access. This was highlighted through the evaluation process and is consistent with previous research which has shown the importance of maintaining widespread engagement and consultation to improve health care delivery [16].

Engagement with health care services by Aboriginal and Torres Strait Islander Peoples is essential to reducing the current health disparities experienced by this population [16, 25]. While we have shown a lack of documented outcomes for foot care services for Aboriginal and Torres Strait Islander Peoples, there are a number of examples of new services that have improved access to general health care and diabetes care services resulting in improved health outcomes in Aboriginal and Torres Strait Islander Communities in Australia [9, 26–28]. Similar to the Buridja Clinic, these share common characteristics, including Community consultation in the development, implementation and ongoing management of the service, involvement of Aboriginal Health Workers, and a focus on self-management and patient participation in health through improved health literacy [26–28]**.** The results of this present study identified issues related to physical clinic access via transport and restricted operating hours which are consistent with previous findings in cancer patients [29] and remain a challenge for the Buridja Clinic to fully address. Options to improve access, including sourcing transport from community-based services or family or Community members, and consideration of alternating the day of the week that the service is provided may be considered as part of the ongoing evaluation of the Buridja Clinic. However, there was also consensus among participants that they were comfortable with the clinic environment indicating it had largely overcome any mistrust of mainstream health care and hospital and clinical environments [29].

Participants also reported clinic attendance improved their knowledge of effective foot care and diabetes management which is significant for reducing the prevalence and impact of DFD. Although this was not measured for this present study and relied on self-report data, it does suggest that this may be achievable with a culturally safe clinic and should be investigated in the future. There is strong evidence that preventative care and education can significantly decrease rates of DFD in the general population [30]. Early mitigation of modifiable risk factors such as smoking, poor diet, alcohol consumption, hypertension and dyslipidaemia while maintaining appropriate blood glucose control is known to reduce risk of future complications [30]. Despite this, prevalence of modifiable risk factors among Aboriginal and Torres Strait Islander Peoples remains high. Other strategies include patient education, implementation of foot checks, objective measures of neurological and vascular health, and early referral to a podiatrist. Again, reported evidence indicates that interaction with early intervention programs and therefore opportunities to educate Aboriginal and Torres Strait Islander cohorts remains low across regions where rates of amputation are high [10, 31]. With Aboriginal and Torres Strait Islander Peoples experiencing lower limb amputation rates up to 38 times higher than those of their age matched, non-Indigenous counterparts, and 98% of lower limb amputations in the population the result of diabetes, the potential impacts of culturally safe, effective foot care education programs to support them to achieve a reduction in  rates of DFD are significant [5, 31, 32].

The findings of this research need to be considered in light of the extent of the data collection and the geographical area in which the study was undertaken. The purpose of the study was to identify strengths and weakness of the clinic service model from a First Nations' perspective. Clients attending the Buridja Clinic volunteered for the research yarns included in this study. Most participants had attended on a number of occasions suggesting they were happy and engaged with the service. Nevertheless determining why they were comfortable with the service is essential to being able to translate this service to other regions. As none of the participants had only attended on a single occasion it is likely that negative feedback regarding the clinic was missed in this aspect of that data collection. In addition, the research yarns were led by an Aboriginal researcher also involved in the clinic. Although this may have introduced bias in the responses based on a Western approach to research methodology, we consider this a strength of this study. The researcher is a member of the local Community, Community members trust and respect this researcher and, combined with the Community ownership of the clinic, this created a safe space for exchange of ideas and criticism that was supported by the inclusion of Elders in each yarning circle [33, 34].

The data collected through anonymous surveys was more likely to have captured negative experiences, however, the overall positive feedback through this method suggests strong dissatisfaction with the clinic is unlikely. Of note, approximately 25% of the total number of clients attending the clinic completed a survey. Completion, although supported by an Aboriginal Health Worker, may have been affected by the written nature of the survey and the feedback and client comfort and confidence with undertaking this activity. As previously discussed, research yarns are a more culturally appropriate methodology for determining participant and Community perspectives. In this instance, this aspect of the study would have potentially been strengthened with additional research yarns undertaken in the Community in addition to those undertaken with clinic clients. The feedback in relation to the Buridja Clinic is specific to the Aboriginal and Torres Strait Islander Community on the Central Coast of New South Wales. It is well established that consulting with Aboriginal and Torres Strait Islander Communities and services in the establishment of new health care services is vital. However, services also need to be tailored to specific needs and preferences of individual Communities, as there are cultural differences between Communities. Therefore, while specific elements of the Buridja Clinic that worked well including design aspects and inclusion of students were identified in this study, implementation of these elements into other Communities would require prior Community consultation and engagement.

## Conclusion

Evaluation of a foot care service for Aboriginal and Torres Strait Islander people embedded in a university-based podiatry program demonstrated the incorporation of specific service design elements, including yarning circles and group appointments, as well as, student placements encouraged ongoing Community engagement with the service. Participants reported improved foot health, greater foot and self-care knowledge, and overall better general health and management as a result of attendance to the clinic. Consideration needs to be given to addressing limited access to transport and flexible operating hours when establishing similar services.

## Supplementary Information

**Additional file 1.**

## Data Availability

Requests for further detail on the data collected in this study, or data sharing arrangements, can be submitted to Vivienne Chuter (Vivienne.chuter@newcastle.edu.au).

## Abbreviations

DFD: Diabetes-related foot disease
NAIDOC: National Aborigines and Islanders Day Observance Committee
SD: Standard deviation
UON: University of Newcastle
