# Supplementary material for: Yarning about foot care: evaluation of a foot care service for Aboriginal and Torres Strait Islander Peoples
Source: J Foot Ankle Res. 2022 Apr 4;15:25. doi: 10.1186/s13047-022-00524-9 (PMC8978452; doi:10.1186/s13047-022-00524-9)
Supplement: Supplementary file 1 — Additional file 1. [file 13047_2022_524_MOESM1_ESM.docx]

**The Buridja Clinic… client feedback survey**

Thank you for attending the Buridja Clinic. The overarching aim of the clinic is to provide a service that helps you to care for your feet and, to prevent foot complications as a result of diabetes. To help us to continue to improve the clinic we would greatly appreciate you answering the following questions:

1. **Approximately how many times have you attended the clinic?** _____________
2. **How did you find out about the service?** (please circle)

Referral from another health service

Advertising

Word of mouth

Other (please specify)

__________________________________________________________________________________________________________________________________________________

1. **Which of the following clinic services have you accessed?** (please circle)

Diabetes assessment

General foot care

Arch supports

Nail surgery

Footwear referral

Diabetes education session

Other (please specify)

__________________________________________________________________________________________________________________________________________________

1. **How easy did you find it to access the service?** This includes making an appointment and getting to the clinic. (Please circle)

Very easy

Easy

Neutral

Difficult

Very difficult

**Do you have any suggestions for how we could make accessing the clinic easier for you?**

__________________________________________________________________________________________________________________________________________________

1. **How would you rate the availability of foot care services in the clinic?** (Please circle)

Very good

Good

Neutral

Poor

Very poor

**Do you have any suggestions for additional foot care services you would like us to offer?**

__________________________________________________________________________________________________________________________________________________

1. **How would you rate your experience with the students in the clinic?** (Please circle)

Very good

Good

Neutral

Poor

Very poor

**Do you have any suggestions for how we can improve your experience with the students?**

__________________________________________________________________________________________________________________________________________________

1. **How would you rate your experience with the staff in the clinic?** (Please circle)

Very good

Good

Neutral

Poor

Very poor

**Do you have any suggestions for how we can improve your experience with the staff?**

__________________________________________________________________________________________________________________________________________________

1. **How would you rate the care you received in the clinic?** (Please circle)

Very good

Good

Neutral

Poor

Very poor

**Do you have any suggestions for how we can improve the care we offer in the clinic?**

__________________________________________________________________________________________________________________________________________________

1. **How likely are you to recommend the clinic services to your friends and family?**  (Please circle)

Very likely

Likely

Neutral

Unlikely

Very Unlikely

1. **Do you have any other feedback that might improve the clinic?**

____________________________________________________________________________________________________________________________________________

**Thank you for providing us with your feedback. Your responses are important to us and will be used to help improve the service in the future.**
